# Phytochemical Analysis, Antioxidant, Antistress, and Nootropic Activities of Aqueous and Methanolic Seed Extracts of Ladies Finger (*Abelmoschus esculentus* L.) in Mice

**DOI:** 10.1155/2014/519848

**Published:** 2014-10-21

**Authors:** Sathish Kumar Doreddula, Srinivasa Reddy Bonam, Durga Prasad Gaddam, Brahma Srinivasa Rao Desu, Nadendla Ramarao, Vijayapandi Pandy

**Affiliations:** ^1^Department of Pharmacology, Chalapathi Institute of Pharmaceutical Sciences, Guntur, Andhra Pradesh 522 034, India; ^2^Vaccine Immunology Laboratory, Natural Product Chemistry Division, CSIR-Indian Institute of Chemical Technology, Tarnaka, Hyderabad 500007, India; ^3^Department of Pharmaceutical Sciences, Birla Institute of Technology, Mesra, Ranchi, Jharkhand 835215, India; ^4^Center for Chemical Biology, CSIR-Indian Institute of Chemical Technology, Tarnaka, Hyderabad 500007, India; ^5^Department of Pharmacology, Faculty of Medicine, University of Malaya, 50603 Kuala Lumpur, Malaysia

## Abstract

*Abelmoschus esculentus* L. (ladies finger, okra) is a well-known tropical vegetable, widely planted from Africa to Asia and from South Europe to America. In the present study, we investigated the *in vitro* antioxidant capacity and *in vivo* protective effect of the aqueous and methanolic seed extracts of *Abelmoschus esculentus* against scopolamine-induced cognitive impairment using passive avoidance task and acute restraining stress-induced behavioural and biochemical changes using elevated plus maze (EPM) and forced swimming test (FST) in mice. Our results demonstrated that the pretreatment of mice with aqueous and methanolic seed extracts of *Abelmoschus esculentus* (200 mg/kg, p.o.) for seven days significantly (*P* < 0.01) attenuated scopolamine-induced cognitive impairment in the passive avoidance test. In addition, these extracts significantly reduced the blood glucose, corticosterone, cholesterol, and triglyceride levels elevated by acute restraint stress and also significantly increased the time spent in open arm in EPM and decreased the immobility time in FST. It has also been revealed that these extracts showed a significant antioxidant activity and no signs of toxicity or death up to a dose of 2000 mg/kg, p.o. These results suggest that the seed extracts of *Abelmoschus esculentus* L. possess antioxidant, antistress, and nootropic activities which promisingly support the medicinal values of ladies finger as a vegetable.

## 1. Introduction

Stress is “a condition or feeling experienced when a person perceives that demands exceed the personal and social resources that the individual is able to mobilize.” In our day-to-day life, every human faces a stressful situation which is characterized by a combination of physiologic, neuroendocrine, behavioural, and emotional responses to novel or threatening stimuli. Stress causes disturbance in the body's normal physiological equilibrium and results in threatened homeostasis and there is increasing evidence that overstress affects cognitive functions and contributes to the development of many neuropsychiatric and neurodegenerative disorders including depression and anxiety, Alzheimer's disease, and Parkinson's disease [1]. Depression and anxiety disorders are extremely common in general population [2]. Stress response activates body's hypothalamic-pituitary-adrenal (HPA) axis resulting in elevated corticosterone hormone levels. Previous studies have shown that acute restraint stress in rodents increases anxiety and depression-like behaviours [3]. An animal model that generates depression and anxiety-like behaviours in response to stressful stimuli is very useful in determining antidepressant and anxiolytic efficacy. The elevated plus maze (EPM) is a well-established model for studying anxiety-like behaviour and the immobility of the animal in forced swimming test (FST) reflects a state of “behavioural despair” used to evaluate depression-like behaviour [2, 4]. Restraint stress or immobilization has been used extensively as a stressor for the study of stress-related biological, biochemical, and physiological responses in animals and is commonly used because it is less severe than other physical stressors, such as a foot shock, but is still capable of activating the stress response. In this type of stressor, movement is limited by placement in a Plexiglas chamber or immobilization bag.

Dementia is a mental chronic disorder characterized by the loss of intellectual ability, sufficiently severe as to involve impairment of memory. The most common cause of dementia is Alzheimer's disease, which is a progressive neurodegenerative disorder associated with loss of neurons in brain specific areas especially in the hippocampus [5]. The central cholinergic system plays an important role in learning and memory processes. Centrally acting antimuscarinic drugs such as scopolamine impairs learning and memory both in animals and in human beings. Dementia is largely a hidden problem as per the epidemiological studies in India. Prevalence rates for dementia increase exponentially with advancing age. Since allopathic system of medicine is yet to provide a radical cure, it is worthwhile to look into new directions, which would minimize the incidents of memory loss seen in elderly patients [6].

Medicinal plants are important sources of biologically active antioxidants. Natural antioxidants, which are ubiquitous in fruits and vegetables, have also received great attention and have been studied extensively, since they are effective free radical scavengers and are assumed to be less toxic than synthetic antioxidants [7]. Green leafy vegetables provide a high amount of carotene, ascorbic acid, and microelements which play important roles in nutrient metabolism and slowing down of degenerative diseases [8].* Abelmoschus esculentus* L. (Family: Malvaceae), also known as* Hibiscus esculentus*, is an important vegetable, widely distributed from Africa to Asia, Southern Europe, and America that is more commonly known as ladies finger, okra, or gumbo [9]. The fibres in ladies finger help to stabilize blood sugar by regulating the rate at which sugar is absorbed from the intestinal tract. Previous studies reported that ladies finger polysaccharide possesses hepatoprotective [10], antidiabetic [11], antiulcer [12], anticancer [13, 14], anti-inflammatory, laxative, antihyperlipidemic, antifungal, and analgesic activities [15]. Recently, some quercetin derivatives, well-known antioxidants, were identified and isolated from ladies finger [16]. Nutritionally, the richest part of the ladies finger plant is the dried seeds. The oil of ladies finger seeds is edible and the residual meal after oil extraction is rich in protein. With this background, the present investigation aims at exploring the natural antioxidant, antistress, and nootropic activities of the aqueous and methanolic seed extracts of* Abelmoschus esculentus* (AE, ME).

## 2. Materials and Methods

### 2.1. Chemicals and Reagents

Diazepam and piracetam were obtained from Cipla Ltd., Mumbai, India. *β*-Carotene, linoleic acid, and butylated hydroxytoluene (BHT) were purchased from Hi-Media (Mumbai, India). DPPH (2,2-Diphenyl-1-(2,4,6-trinitrophenyl)hydrazyl) and (-)-scopolamine hydrobromide trihydrate were purchased from Sigma-Aldrich (St. Louis, MO, USA). TLC-solvents were of HPLC grade obtained from Merck, Mumbai, India. All remaining reagents were used of analytical grade.

### 2.2. Plant Material

The seeds of* Abelmoschus esculentus* were collected from Nallaguntla, Prakasam, Andhra Pradesh (A.P.), India. The seeds were identified and authenticated by Dr. Khasim, Head of Department of botany, Acharya Nagarjuna University, Guntur, A.P., India, and a copy of voucher specimen was deposited in the herbarium of the Department of Pharmacognosy, Chalapathi Institute of Pharmaceutical Sciences, Guntur, A.P., India, for the future reference. After due authentication, seeds were cleaned and shade dried at 30 ± 5°C and pulverized using a mechanical grinder. The coarsely ground powder was passed through sieve number 40 and stored in an airtight container at −20°C until extraction.

### 2.3. Preparation of Plant Extracts

#### 2.3.1. Methanolic Extract of* Abelmoschus esculentus* Seeds (ME)

The powdered material (500 g) in 80% v/v methanol was sonicated for 1 h and then extracted with 80% v/v methanol in a Soxhlet extractor (1000 mL) for 72 h at room temperature with constant stirring. The extract was filtered and the filtrate was concentrated at 30°C under reduced pressure in a rotary evaporator until a crude solid extract was obtained which was then freeze-dried for complete solvent removal. The percentage yield obtained was 13% w/w.

#### 2.3.2. Aqueous Extract of* Abelmoschus esculentus* Seeds (AE)

The 500 g seed powder was macerated and extracted with distilled water for 72 h at room temperature. Chloroform was added to the mixture to prevent microbial contamination. After completion of extraction, it was filtered and the solvent was removed by evaporation in a rotary evaporator and the solid mass was freeze-dried. The percentage yield obtained was 17% w/w.

### 2.4. Phytochemical Screening

Preliminary phytochemical tests for the detection of carbohydrates, phenols, flavonoids, alkaloids, terpenoids, steroids, tannins, saponins, and cardiac glycosides were conducted using standard phytochemical methods as described elsewhere [17].

#### 2.4.1. Thin Layer Chromatography (TLC) Analysis

AE and ME were loaded on silica plate (Merck Aluminium sheet—silica gel 60 F 254). A mixture of hexane : chloroform : methanol (8 : 2 : 1) was used as the solvent system. The TLC plate was kept in iodine chamber for one minute and under UV light (254 nm) to visualize bands on chromatogram [18, 19].

#### 2.4.2. Fourier Transform Infrared (FTIR) Fingerprint Analysis

Fourier transform infrared (FTIR) spectrophotometer was used to identify the characteristic functional groups in the seed extracts. The AE and ME (5 mg), respectively, were thoroughly mixed with potassium bromide (KBr) in a mortar and pressed at pressure of 6 bars within 2 min in order to prepare a thin translucent sample discs. The FT-IR spectrum was obtained using Perkin Elmer 2000 spectrophotometer system with a scan range from 400 to 4000 cm^−1^ and analysed using Bruker OPUS software [19–21].

### 2.5. *In Vitro* Antioxidant Activity

#### 2.5.1. Ferric Reducing Antioxidant Power (FRAP) Assay

Ferric-reducing antioxidant power of AE and ME was measured by the direct reduction of Fe^3+^(CN)_6_ to Fe^2+^(CN)_6_ and was determined by measuring absorbance resulting from the formation of the Perl Prussian blue complex following the addition of excess ferric ions (Fe^3+^) [22, 23]. Briefly, various concentrations of AE and ME (2.5 mL) were mixed with 2.5 mL of 200 mM sodium phosphate buffer (pH 6.6) and 2.5 mL of 1% w/v potassium ferricyanide [K_3_Fe(CN)_6_]. The mixture was incubated at 50°C for 20 min and then 2.5 mL of 10% v/v trichloroacetic acid was added. This mixture was centrifuged at 1000 rpm for 10 min. The supernatant (5 mL) was mixed with an equal volume of deionized water and 0.5 mL of 0.1% w/v ferric chloride and the absorbance was measured spectrophotometrically at 700 nm. The assays were carried out in triplicate and the results are expressed as mean ± standard error. Increased absorbance of the reaction mixture indicates greater reduction capability [24].

#### 2.5.2. DPPH Radical Scavenging Assay

The stable 1,1-diphenyl-2-picryl hydrazyl (DPPH) free radical scavenging activity of the AE and ME was determined by the method described by Blois, with slight modification [25]. One mL of 0.2 mM DPPH solution in methanol was mixed with the 1 mL extracts of 125, 250, 500, 1000, and 2000 *µ*g/mL concentrations, respectively. The mixture was incubated in dark for 20 min at room temperature and the absorbance was measured at 517 nm. The free radical scavenging activity of AE and ME was determined by comparing its absorbance with that of a blank solution (no sample). Butylated hydroxytoluene (BHT) was used as a reference standard. The ability to scavenge the DPPH radical was calculated using the following equation:

(1) %  of  DPPH  radical  scavenging  activity =(A0−A1)A0×100,

where *A*_0_ is the absorbance of the blank and *A*_1_ is the absorbance of the sample or standard.

#### 2.5.3. *β*-Carotene-Linoleic Acid Assay

Antioxidant activity was determined by measuring the inhibition of volatile organic compounds and the conjugated diene hydroperoxides arising from linoleic acid oxidation described by Wettasinghe and Shahidi [26]. One mL of *β*-carotene solution (0.2 mg/mL in chloroform) was pipetted into a round-bottom flask containing 0.02 mL of linoleic acid and 0.2 mL of Tween 20. The mixture was then evaporated at 40°C for 10 min using a rotary evaporator to remove chloroform. Then, 100 mL of oxygenated distilled water was added slowly, with vigorous shaking to form an emulsion. 5 mL aliquots of the emulsion were transferred into different test tubes containing 0.2 mL of various concentrations (0.5–20.0 mg/mL) of AE and ME in methanol and the mixture was then gently mixed and placed in a water bath at 50°C for 2 h. The same procedure was repeated with the positive control BHT and a blank. The absorbance of the mixtures was measured at 470 nm using a spectrophotometer until the *β*-carotene colour disappeared. All determinations were performed in triplicate.

The *β*-carotene bleaching rate (*R*) was calculated according to

(2) $$\begin{array}{c} R\;=\ln\frac{\left(a/b\right)}{t}, \end{array}$$

where ln⁡ = natural log, *a* = absorbance at time *t*(0), and *b* = absorbance at time (120 min).

The total antioxidant activity (AA) was calculated as the percent inhibition relative to the control using

(3) $$\begin{array}{c} \text{AA}=\left[\frac{\left(R_{\text{control}}-R_{\text{sample}}\right)}{R_{\text{control}}}\right]\times 100. \end{array}$$

#### 2.5.4. Chelating Effects on Ferrous Ions

The chelating effect was determined according to the method of Dinis et al., [27]. Briefly, 0.1 mL of various concentrations (0.063–1.0 mg/mL) of AE and ME in methanol was added to 0.1 mL of 2 mM FeCl_2_. The reaction was initiated by adding 0.2 mL of 5 mM ferrozine (3-[2-Pyridyl]-5, 6-diphenyl-1, 2, 4-triazine-4, 4′-disulfonic acid sodium-salt) and the mixture was incubated at 37°C for 10 min. After adding 1.5 mL of double distilled H_2_O to the mixture, the absorbance was measured at 562 nm. Ultrapure water instead of Ferrozine solution was used as a blank and BHT was used as reference standard. The inhibition percentage of the ferrozine-Fe^2+^ complex formation was calculated using the following formula:

(4) Ferrous  ion-chelating  ability(%) =[(Acontrol−Asample)Acontrol]×100,

where *A*_control_ is the absorbance of the control (control contained FeCl_2_ and ferrozine; complex formation molecules) and *A*_sample_ is the absorbance of the test compound.

### 2.6. Experimental Animals

Adult male Swiss albino mice (22 ± 2 g) were housed under standard laboratory conditions of temperature (22 ± 3°C), humidity (60 ± 5%), and illumination (12-h light/dark cycle) in polypropylene cages and were fed water and standard laboratory food* ad libitum*. The mice were initially acclimatized to the experimental housing conditions and animal handlers for 3 days prior to all experiments in order to minimize handling stress during the test. Healthy mice were selected based on the swimming ability, rotarod test, and normal social behaviour. All experiments were conducted in accordance with National Guidelines (CPCSEA) on the* Proper Care and Use of Animals in Laboratory Research* (Indian National Science Academy, New Delhi, 2000) and were approved by the Institutional Animal Ethics Committee (IAEC) (Approval number 6/IAEC/CPS/M.PHARM/2013-14).

### 2.7. Acute Toxicity Study

Acute oral toxicity of* Abelmoschus esculentus* L. seed extracts was determined as per Organization for Economic Cooperation and Development (OECD) guidelines 423 [28]. A single oral dose (5, 50, 300, and 2000 mg/kg) of extracts was administered to four mice groups (*n* = 6) and was observed individually at least once during the first 30 min, periodically during the first 24 h, with special attention given during the first 4 hours and daily thereafter, for a total of 14 days for abnormal signs, diarrhea, and food and water intake. The animal body weight and locomotor activity score using actophotometer were measured at 0th (initial) and 14th (final) days. On the 14th day, the mice were anesthetized through intraperitoneal injection of a cocktail containing ketamine (80 mg/kg) and xylazine (10 mg/kg). Blood samples were collected by cardiac puncture and hematological parameters were analysed using ABX Micros-60 hematology analyser. All the vital organs were collected and weighed. The heart, liver, and kidney of the mice treated with AE and ME (2000 mg/kg, p.o) were processed for haematoxylin and eosin (H&E) histopathological staining. Mice did not show any signs of toxicity or death up to a dose of 2000 mg/kg; hence, 1/10th of the dose 200 mg/kg was taken as an effective dose for* in vivo* pharmacological studies [29, 30].

### 2.8. Experimental Design

The mice were randomly divided into two sets each with five groups (*n* = 6). First set of animals were divided as Group I: control (saline), Group II: negative control (scopolamine), Group III: positive control (scopolamine + piracetam), Group IV: scopolamine + aqueous extract (AE), and Group V: scopolamine + methanolic extract (ME) used for evaluation of nootropic activity. Another set was divided into Group I: saline control (not exposed to stress); Group II: negative control (untreated stress-induced); Group III: positive control (diazepam/imipramine treated stress-induced); Group IV: aqueous extract (AE) + stress-induced; Group V: methanolic extract (ME) + stress-induced used for antistress screening. The aqueous and methanolic extracts (200 mg/kg, p.o.) or piracetam (200 mg/kg, i.p.) were administered once daily for seven days. Memory impairment was induced by scopolamine treatment (0.4 mg/kg, i.p.) 30 min before the passive avoidance task. Diazepam (2 mg/kg, i.p.) or imipramine (12 mg/kg, i.p.) was given 30 min before the stress induction.

### 2.9. Evaluation of Nootropic Activity

#### 2.9.1. Passive Avoidance Task

Passive avoidance task was carried out as described elsewhere [31, 32]. The apparatus (Gemini Avoidance System, San Diego, CA, USA) consisted of equally sized light and dark compartments (20 cm × 20 cm × 20 cm) separated by a guillotine door (5 cm × 5 cm). The illuminated compartment contained a 50 W bulb, and the floor of nonilluminated compartment was composed of 2 mm stainless steel rods spaced 1 cm apart. Mouse was gently placed in the illuminated compartment for the acquisition trial, and the door between the two compartments was opened 10 s later. When the mouse entered the dark compartment, the door automatically closed and an electrical foot shock (0.5 mA) of 2 s duration was delivered through the stainless steel rods. Twenty-four hours after this acquisition trial, the mouse was again placed in the illuminated compartment for a retention trial. The time taken for a mouse to enter the dark compartment after its door was opened was defined as step-down latency (SDL) for both acquisition and retention trials. Latency for entering the dark compartment was recorded up to 300 s. If a mouse did not enter the dark compartment within 300 s, the mouse was removed and assigned a latency score of 300 s.

### 2.10. Evaluation of Anti-Stress Activity

#### 2.10.1. Acute Restraint Stress

Acute restraint stress model was used according to Masood et al., with minor modifications [33]. All the animals were pretreated with the respective vehicle or extract or standard drug for 7 days before the induction of stress. After 30 min of pretreatment on day 7, mice were exposed to stressful stimuli induced by restraining the animals in polyvinyl chloride (PVC) restrainers of 105 mm length and 32 mm diameter for a period of 4 hours. Following the induction of stress, the mice were evaluated for behavioural changes using the EPM and FST. A different set of mice treated as above and the blood was withdrawn from the retro orbital plexus and plasma corticosterone, glucose, total protein, cholesterol, and triglycerides were measured [34].

#### 2.10.2. Elevated Plus Maze Task (EPM)

EPM is a validated behavioural assay for measuring anxiolytic and anxiogenic-like activities of pharmacological agents in rodents [35]. In brief, the apparatus consisted of two opposite open arms (30 × 5 cm) and two enclosed arms (30 × 5 × 15 cm) extending from a common central platform (5 × 5 cm). The maze was 40 cm above the floor and was constructed from plexiglass. Mice were placed individually onto the center of the apparatus facing an open arm. The time spent in each arm and the number of entries into each arm were manually recorded by blind observer for 5 min. After each trial, the maze was wiped clean with 10% v/v ethanol solution and dried. An entry was defined as all four paws having crossed the line between the arm and the central area. Anxiolytic action was defined by increased time in and/or number of entries to open arms. The entries in closed arms and total entries were used to reflect the motor component of exploratory activity.

#### 2.10.3. Forced Swimming Test (FST)

FST was carried out according to the method described by Deepika et al., with slight modifications [36, 37]. The mice were individually placed into transparent plexiglass cylinders (25 cm height × 18 cm in diameter) filled with water to a 15-cm depth at 25°C. Each mouse was placed into the water and forced to swim for 6 min. The total duration of immobility (*s*) was recorded during the last 4 min of a single 6-minute test session. Mice were considered immobile when they made no attempts to escape with the exception of the movements necessary to keep their heads above the water.

### 2.11. Statistical Analysis

The results are expressed as the mean ± S.E.M. The data were analysed for statistically significance using two-way analysis of variance (ANOVA) followed by* Post hoc* multiple comparisons using Bonferroni test for antioxidant assays and passive avoidance test. One-way analysis of variance (ANOVA) followed by* Post hoc* multiple comparisons using Tukey test was performed for plasma parameters, elevated plus maze test, and forced swimming test. *P* < 0.05 was considered to be statistically significant.

## 3. Results

### 3.1. Phytochemical Screening

Preliminary phytochemical studies of AE and ME revealed the presence of alkaloids, carbohydrates, flavonoids, phenols, proteins, terpenoids, tannins, and sterols. Saponins and cardiac glycosides were found to be absent in AE and ME.

#### 3.1.1. Thin Layer Chromatography Profiling

**Figure 1:**
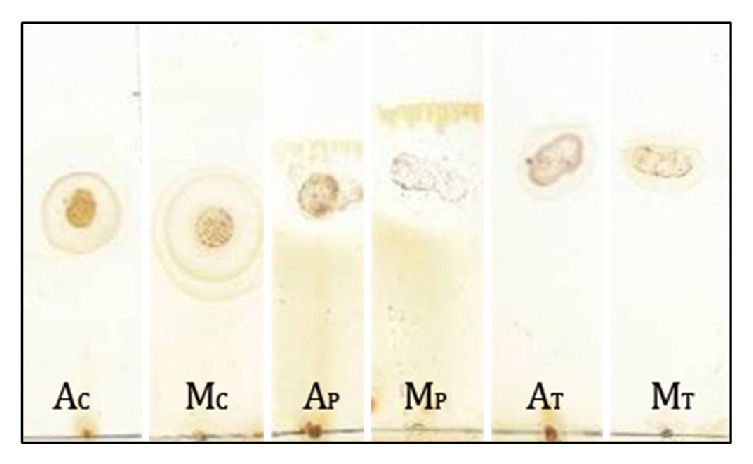
Thin layer chromatogram of* Abelmoschus esculentus* seed extracts. A: aqueous extract, M: methanolic extract. The extracts were chromatographed in solvent system containing hexane : chloroform : methanol (8 : 2 : 1) followed by spraying of visualization reagents for carbohydrates (AC&MC), phenols (AP&MP), and terpenoids (AT&MT).

Several bands were observed during partitioning of extract components with solvent system indicating separation of phytoconstituents depending on polarity. Carbohydrates, phenols, and terpenoids were identified on chromatogram after spraying specific detecting agents as shown in Figure 1. The *R*_*F*_ values of carbohydrates, phenols, and terpenoids were found to be 0.68, 0.70, and 0.85 for aqueous extract and 0.65, 0.73, and 0.84 for methanolic extract respectively.

#### 3.1.2. Fourier Transform Infrared Fingerprint Analysis

**Figure 2:**
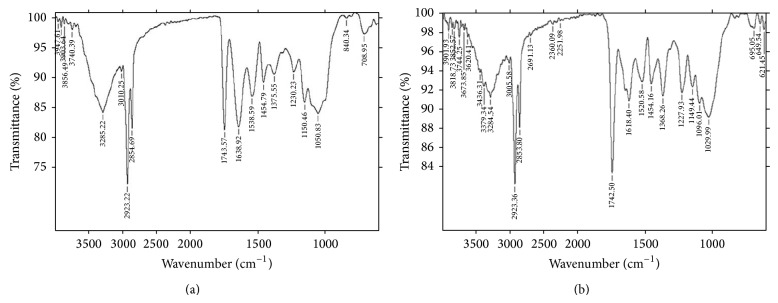
FTIR spectrum of (a) aqueous, (b) methanolic seed extract of* Abelmoschus esculentus.*

FTIR spectroscopic studies revealed the presence of various functional groups such as alkyl, ketone, aldehyde, carboxylic acids, esters, and amide in aqueous and methanolic extracts of* Abelmoschus esculentus*, respectively (Table 1 and Figures 2(a) and 2(b)).

### 3.2. Antioxidant Activity

#### 3.2.1. Ferric Reducing Antioxidant Power (FRAP) Assay

**Figure 3:**
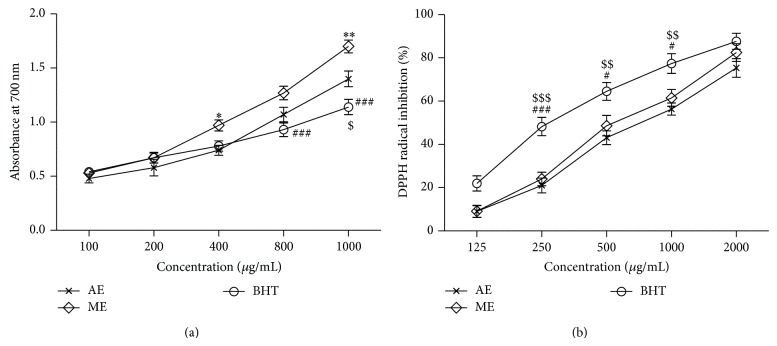
(a) Ferric reducing power, (b) DPPH radical scavenging activity of BHT, AE, and ME seed extracts of* Abelmoschus esculentus*. Data are representative of three independent experiments. Values are mean ± S.E.M. The significance of differences was analysed by two-way ANOVA followed by Bonferroni multiple comparisons test (AE versus ME, ^*^ *P* < 0.05; ^**^ *P* < 0.01; ME versus BHT, ^#^ *P* < 0.05; ^###^ *P* < 0.001; AE versus BHT, ^$^ *P* < 0.05; ^$$^ *P* < 0.01; ^$$$^ *P* < 0.001). (a) Treatment: F (2, 30) = 20.62, *P* < 0.0001; concentration: F (4, 30) = 122.39, *P* < 0.0001; interaction (treatment × concentration): F (8, 30 = 4.79, *P* < 0.001). (b). Treatment: F (2, 30) = 35.82, *P* < 0.0001; concentration: F (4, 30) = 158.48, *P* < 0.0001; interaction (treatment × concentration): F (8, 30 = 1.12, n.s).

The reducing power of aqueous (AE) and methanolic (ME) seed extracts of* Abelmoschus esculentus* compared with reference BHT in FRAP assay has been shown in Figure 3(a). The reducing power of these extracts was observed in a dose-dependent manner. The maximum reducing power of these extracts was determined at 1 mg/mL.

#### 3.2.2. DPPH Radical Scavenging Assay

Free radical scavenging effects of the AE and ME on DPPH radicals increased with increase in concentration. At 0.125 to 2.0 mg/mL, the scavenging activities of AE and ME on DPPH radical ranged from 9.05 to 75.35% and 9.11 to 82.42%, respectively (Figure 3(b)). Similarly, the reference BHT (0.12 to 2.0 mg/mL) showed significant free radicals scavenging activities dose-dependently ranging from 21.99 to 87.66%.

#### 3.2.3. Antioxidant Activity against *β*-Carotene-Linoleic Acid

**Figure 4:**
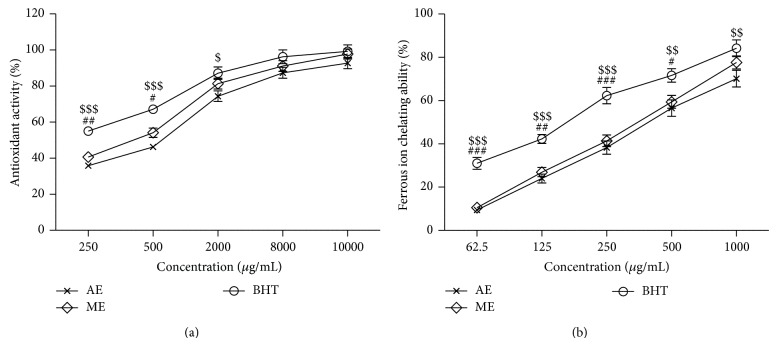
(a) % Antioxidant activity using *β*-carotene bleaching rate. (b) Chelating effect on ferrous ion, of BHT, AE, and ME seed extracts of* Abelmoschus esculentus*. Data are representative of three independent experiments. Values are mean ± S.E.M. The significance of differences was analysed by two-way ANOVA followed by Bonferroni multiple comparisons test (AE versus ME, ^*^ *P* < 0.05; ^**^ *P* < 0.01; ME versus BHT, ^#^ *P* < 0.05; ^###^ *P* < 0.001; AE versus BHT, ^$^ *P* < 0.05; ^$$^ *P* < 0.01; ^$$$^ *P* < 0.001). (a) Treatment: F (2, 30) = 29.02, *P* < 0.0001; concentration: F (4, 30) = 195.43, *P* < 0.0001; interaction (treatment × concentration): F (8, 30 = 1.51, n.s). (b) Treatment: F (2, 30) = 58.46, *P* < 0.0001; concentration: F (4, 30) = 207.34, *P* < 0.0001; interaction (treatment × concentration): F (8, 30 = 1.18, n.s).

Figure 4(a) shows the antioxidant activities of the AE and ME compared to BHT. At 0.25 to 10.0 mg/mL, the antioxidant activities of AE, ME, and BHT ranged from 35.84 to 92.76%, 40.68 to 97.76%, and 55 to 99.21%, respectively.

#### 3.2.4. Chelating Effects on Ferrous Ions

The ability of ferrous ion chelating by the AE and ME was increased in a dose-dependent manner, as illustrated in Figure 4(b). The strongest chelating effect (77.60%) was obtained from ME at 1.0 mg/mL. At this concentration, the lowest chelating effect was exhibited by AE (70.11%) and the highest one was exhibited by BHT (84.20%).

### 3.3. Acute Oral Toxicity Study

**Figure 5:**
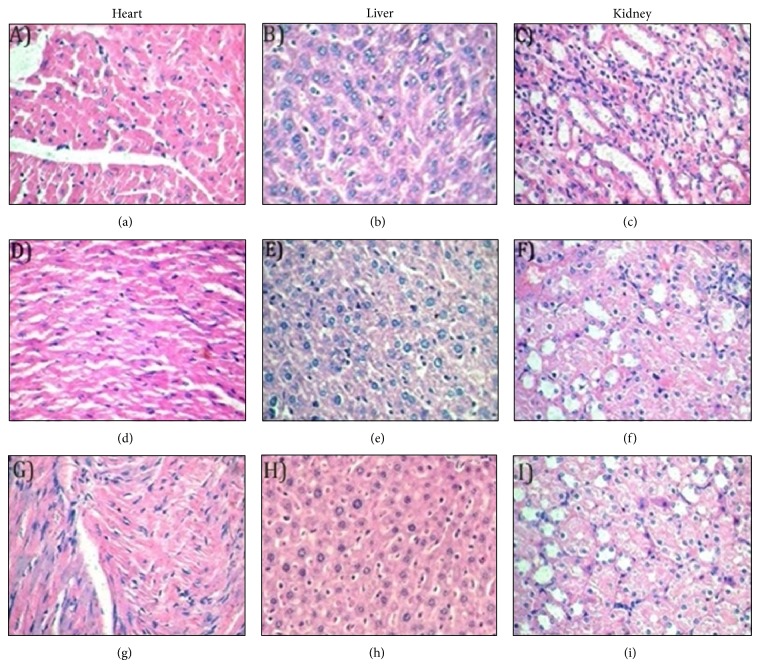
Hematoxylin and eosin (H&E) stained photo micrographs of mice heart, liver, and kidney tissues (20× magnification). (a), (b), and (c) indicate control, (d), (e), and (f); (g), (h), and (i) show no abnormality in mouse group treated with AE and ME (2000 mg/kg, p.o.), respectively.

In the acute oral toxicity study, mice did not show any signs of toxicity up to 2000 mg/kg and hence the LD_50_ cut-off value might exceed 2000 mg/kg. The spontaneous locomotor activity score obtained for 10 minutes when mice were administered with 2000 mg/kg of AE and ME seed extracts, respectively, at 0th day and 14th day was found to be 318.51 ± 2.65; 327.51 ± 2.50 and 325 ± 4.13; 332.36 ± 2.7, respectively. The initial and final body weight of mice treated with 2000 mg/kg of AE and ME was 23.10 ± 1.82; 25.84 ± 1.64 and 23.94 ± 1.76; 24.24 ± 1.93 g respectively. The mean relative organ weights and hematological parameters of mice are shown in Tables 2 and 3, respectively. The H&E histopathological staining of heart, liver, and kidney of mice treated with 2000 mg/kg AE and ME is shown in Figure 5. These studies revealed no significant abnormal changes in AE and ME treated mice when compared to control.

### 3.4. Nootropic Activity

#### 3.4.1. Passive Avoidance Task

**Figure 6:**
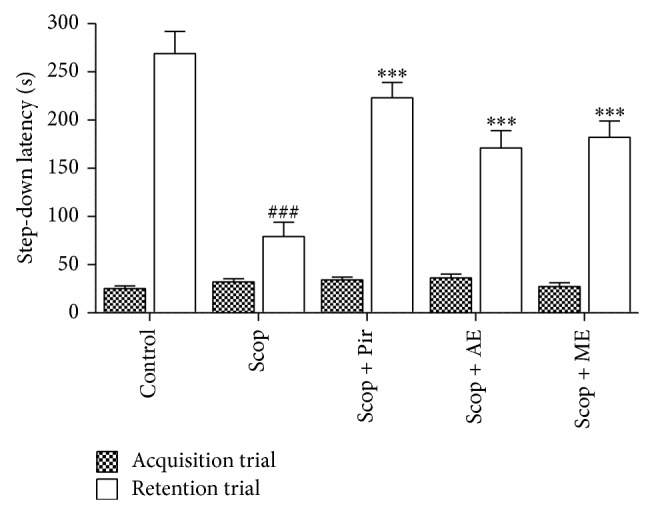
The effect of AE and ME seed extracts of* Abelmoschus esculentus* on scopolamine-induced learning and memory impairment in the passive avoidance test. Control indicates saline-treated group. Scop indicates scopolamine-treated group (0.4 mg/kg, i.p.). Scop + Pir, Scop + AE, and Scop + ME indicate piracetam (Pir), aqueous (AE), and methanolic (ME) seed extracts of* A. esculentus* (200 mg/kg, p.o.), respectively, treated groups for 7 days once daily. Values are mean latency ± S.E.M (*n* = 6). ^###^ *P* < 0.001 when compared to control group and ^***^ *P* < 0.001 when compared to Scop-treated group. Acquisition-retention trials: F (1, 50) = 351.92, *P* < 0.0001; treatments: F (4, 50) = 14.04, *P* < 0.0001; interaction (acquisition-retention trials × treatments): F (5, 50 = 15.68, *P* < 0.0001).

The step-down latency of scopolamine (79.4 ± 15 s) treated mice in the passive avoidance task was significantly (*P* < 0.001) shorter than that of saline control mice (269.02 ± 23 s) as shown in Figure 6. The shorter step-down latency of scopolamine was significantly reversed by pretreatment with reference drug, piracetam (223.2 ± 16.1 s). Similarly, AE (171.01 ± 18.6 s) and ME (182.12 ± 17.5 s) significantly reversed the scopolamine-induced memory impairment (Figure 6).

### 3.5. Antistress Activity

#### 3.5.1. Effect of Seed Extracts of* Abelmoschus esculentus* on Acute Restraint Stress-Induced Changes in Biochemical Parameters

**Figure 7:**
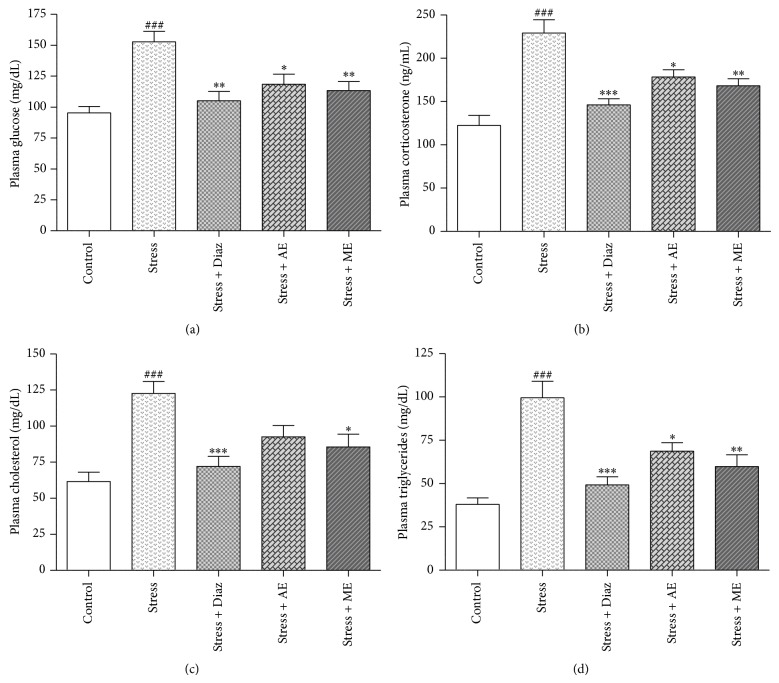
Effect of AE and ME seed extracts of* Abelmoschus esculentus* on acute restraint stress-induced changes in biochemical parameters. (a) Plasma glucose; (b) plasma corticosterone; (c) plasma cholesterol (d); plasma triglycerides. Mice were treated 7 days once daily with diazepam (2 mg/kg, i.p.), AE, and ME (200 mg/kg, p.o.). The values shown are the mean ± S.E.M (*n* = 6); ^###^ *P* < 0.001 when compared to control group, ^*^ *P* < 0.05, ^**^ *P* < 0.01, and ^***^ *P* < 0.001 when compared to stress* per se* group.

The AE and ME showed significant reduction in immobilization stress when compared to the stress* per se* group (Figure 7). The acute restraint stress-induced group showed a marked increase in serum glucose [F (4, 25) = 8.56; *P* < 0.001], corticosterone [F (4, 25) = 14.34; *P* < 0.0001], cholesterol [F (4, 35) = 9.107; *P* < 0.0001], and triglycerides [F (4, 35) = 13.79; *P* < 0.0001] in mice. These stress-induced elevated levels of biochemical parameters were significantly reversed by AE and ME for 7 days once daily and with reference drug, diazepam (2 mg/kg, i.p), pretreatment as shown in Figure 7.

#### 3.5.2. Effect of Seed Extracts of* Abelmoschus esculentus* on EPM

**Figure 8:**
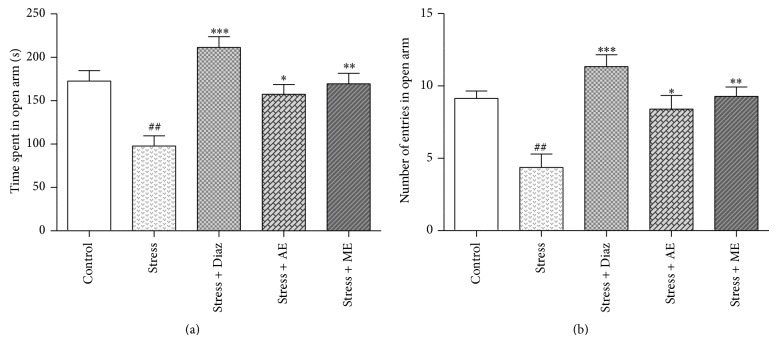
(a) Time spent and (b) number of entries in the open arms in elevated plus maze test. The Diaz, AE, and ME groups were daily treated with the diazepam (2 mg/kg, i.p.), aqueous, and methanolic seed extracts of* Abelmoschus esculentus* (200 mg/kg, p.o.) for 7 days, respectively, once daily and then stress was induced. The values shown are the mean ± S.E.M (*n* = 6); ^#^ *P* < 0.05, ^##^ *P* < 0.01 when compared to control group, ^*^ *P* < 0.05, ^**^ *P* < 0.01, and  ^***^ *P* < 0.001 when compared to stress* per se* group.

The one-way ANOVA results revealed that there was a significant difference between the treatment groups on the time spent [F (4, 25) = 11.80; *P* < 0.0001] and number of entries [F (4, 25) = 10.60; *P* < 0.0001] in the open arms of the elevated plus maze (Figures 8(a) and 8(b)). The stressed mice group spent significantly (*P* < 0.01) less time in the open arms (97.88 ± 11.7 s) when compared to the unstressed control group. Pretreatment with AE and ME, significantly (*P* < 0.05 and *P* < 0.01, resp.) increased the time spent in the open arms (157.31 ± 11.28 s and 169.43 ± 12.07 s, resp.) when compared to the stress* per se* group (97.88 ± 11.70 s). The reference drug, diazepam (2 mg/kg, i.p) also significantly (*P* < 0.001) increased the number of entries and time spent in open arms of the elevated plus maze (Figures 8(a) and 8(b)).

#### 3.5.3. Effect of Seed Extracts of* Abelmoschus esculentus* on the Duration of Immobility in the FST

**Figure 9:**
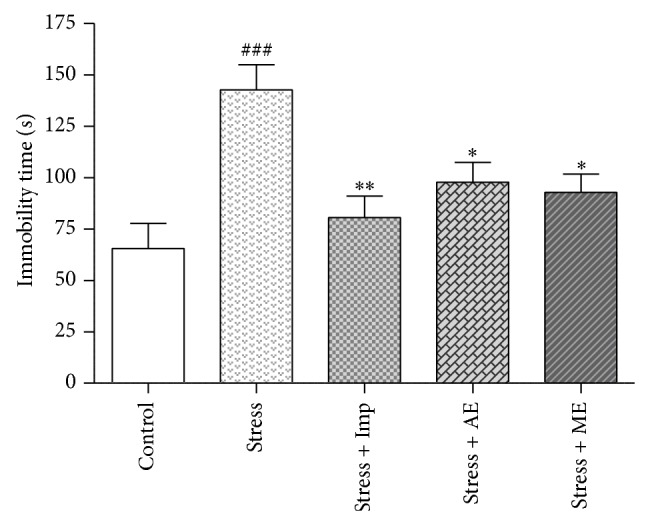
Effect of aqueous and methanolic seed extracts of* Abelmoschus esculentus* on the duration of immobility in the forced swimming test in mice. The Imp, AE, and ME groups were daily treated with imipramine (12 mg/kg, i.p.), aqueous, and methanolic seed extracts (200 mg/kg, p.o.), respectively, for 7 days once daily and then stress was induced after 30 min of last dose. The values shown are the mean ± S.E.M (*n* = 6); ^###^ *P* < 0.001 when compared to control group, ^*^ *P* < 0.05 and ^**^ *P* < 0.01 when compared to stressed group.

The one-way ANOVA results revealed that there was a significant difference between the treatment groups on the immobility time [F (4, 25) = 7.272; *P* < 0.001] in FST. The duration of immobility in stressed mice was significantly (*P* < 0.001) increased when compared to the control group as shown in Figure 9. Pretreatment with AE and ME, significantly (*P* < 0.05) decreased the immobility time (97.80 ± 9.60 s and 92.80 ± 8.90 s, resp.) when compared to the stress* per se* group (142.7 ± 12.2 s). The reference drug, imipramine (12 mg/kg, i.p) also significantly (*P* < 0.01) decreased the immobility time in the forced swimming test (Figure 9).

## 4. Discussion

In worldwide search for novel therapeutic products for the treatment of neurological disorders, ethnopharmacological approach has been progressed constantly, demonstrating the therapeutic effectiveness of different plant species in variety of animal models. Increasing evidences indicate the possible association of oxidative stress with several diseases especially central nervous system disorders including neurodegenerative diseases such as Alzheimer's disease, Parkinson's disease, Down's syndrome, and neuropsychiatric diseases, such as anxiety, schizophrenia, and major depressive disorder [38]. The brain which utilizes 20% of oxygen consumed by the body is highly vulnerable to oxidative stress due to its high O_2_ consumption, modest antioxidant defences, and a high lipid-rich constitution [38]. The lipid-rich constitution of brain favours lipid peroxidation by the reactive oxygen species (ROS) formed primarily by reduction of molecular oxygen from superoxide anions [39, 40].

Natural antioxidants which are ubiquitous in fruits, vegetables, and medicinal plants have received great attention and have been studied extensively, since they are effective free radical scavengers and are assumed to be less toxic than synthetic antioxidants [7]. The present study is a step towards the exploration of natural antioxidant potential, antistress, and nootropic activities of the seed extracts of* Abelmoschus esculentus* as it is an important vegetable and widely consumed across the world.

The antioxidant activity of methanolic seed extracts of* Abelmoschus esculentus* has been studied and reported in the literature [10, 41]. In the present study, the antioxidant activity of aqueous and methanolic seed extracts of* Abelmoschus esculentus* has been compared by using ferric reducing antioxidant power, DPPH radical scavenging, *β*-carotene-linoleic acid assay, and chelating effect on ferrous ions. The IC_50_ values of methanolic seed extract were found to be 542, 416, and 374 *μ*g/mL and for aqueous extract 693, 695, and 437 *μ*g/mL, respectively, in DPPH radical scavenging, *β*-carotene-linoleic acid assay, and chelating effect on ferrous ions. These results revealed that both methanolic and aqueous seed extracts showed very weak antioxidant activity obtained at 1 to 10 mg/mL.

In acute toxicity study, the oral administration of* Abelmoschus esculentus* seed extracts (up to 2000 mg/kg) did not show any signs of toxicity and mortality up to 14 days. Appearance and behaviour of the extracts-treated animals were similar to the vehicle control group during the observation period. Moreover, the spontaneous locomotor activity of the animals treated with extracts AE and ME (up to 2000 mg/kg) was not significantly altered. Spontaneous locomotor activity is an apical test of neuromotor function, representing the peak of neural integration, which has been used for decades to evaluate effects of chemical treatment [42]. In addition, normal body weight gains were observed in the extracts treated groups and there was no significant difference between relative organ weights, haematological parameters, and H&E histopathological staining among treatment groups (Tables 2 and 3; Figure 5). It has been previously reported that potentially toxic substances can drastically reduce the body weight gain and internal organ weights [43]. It has also been demonstrated that the alteration of haematological parameters is a risk evaluation of higher predictive value for human toxicity, when the data are translated from animal studies [44]. Therefore, the present oral acute toxicity study suggest that the aqueous and methanolic seed extracts of* Abelmoschus esculentus* are safe at a dose level of 2000 mg/kg body weight. However, the present study did not evaluate the different vital organs toxicity of the extracts AE and ME using various biochemical parameters. Further acute and subchronic (28 days) oral dose toxicity studies are warranted to investigate the potential toxicity after single and 28-day repeated oral dosing of* Abelmoschus esculentus* extracts in experimental animals.

Stress-induced psychiatric disorders such as anxiety and depression are commonest psychiatric diagnosis in patients attending psychiatric clinics [45]. Approximately two-thirds of the anxious or depressed patients respond to the currently available treatments but the magnitude of improvement is still disappointing. Although there are many effective antidepressants available today, current armamentarium of therapy is often inadequate with unsatisfactory results in about one-third of all subjects treated [46]. Prolonged severe stress creates ineffective adaptation, which results in reduced stamina and mood. The concept of “adaptogens” as a separate group of medicinal substances was first developed by Lazarev in the year 1958 [47]. Since the introduction of adaptogens, several plants that had once been used as tonics have been investigated in Ayurvedic medicine, due to their adaptogenic and rejuvenating properties. The acute restraint stress is a widely used behavioural model to study the molecular basis of stress-related issues [4, 48]. Typically, a stress response is characterized by the activation of hypothalamus-pituitary-adrenal axis resulting in an increase in blood corticosterone levels which in turn lead to an increase in serum triglycerides levels and hyperglycemia [49]. The present study results revealed that administration of* Abelmoschus esculentus* seed extracts (AE and ME) significantly countered the acute restraint stress-induced elevated blood glucose, corticosterone, cholesterol, and triglycerides levels in mice. This demonstrated stress-relieving potential of ladies finger seeds.

EPM and FST are widely used animal models of anxiety and depression, respectively, for the screening of antistress activity of drugs. The FST model is based on despair or helplessness behaviour in response to some inescapable and confined space and is sensitive to various antidepressant drugs. Recently, Ebrahimzadeh et al. demonstrated the antidepressant activity of single dose of* Hibiscus esculentus* methanolic seed and leaf extracts using FST and tail suspension tests (TST) [50]. To further support these results, we have evaluated the antistress potential of seven days pretreatment of aqueous and methanolic seed extracts of* Abelmoschus esculentus* by FST after acute restraint stress induction. The seed extracts (AE and ME) treated mice significantly reversed the acute restraint stress-induced immobility in FST as shown in Figure 9.

The EPM paradigm has been described as a robust method for assessing anxiety responses of rodents. The present study revealed that the acute restraint stressed mice spent significantly less time and less number of entries into the open arms when compared to the control. Whereas mice pretreated with AE and ME significantly increased the time spent and number of entries into open arms, these results further demonstrated the stress-relieving potential of ladies finger seeds.

The loss of cholinergic neurons in the basal forebrain and hippocampus and pharmacological blockade of cholinergic neurons in these areas causes impairment of learning and memory in experimental animals [51, 52]. Several studies have employed scopolamine; a nonselective muscarinic receptor antagonist, used to impair memory in laboratory animals, served as a test model of cognitive function, and this model has been widely used to investigate the memory enhancing properties of new test substances [46, 53–57]. In present study,* Abelmoschus esculentus* seed extracts (AE and ME) were found to be enhancing cognitive function in mouse model of scopolamine-induced amnesia using passive avoidance test. Recently, it has been reported that* Abelmoschus esculentus* L. fruit extract and its bioactive principles (quercetin and rutin) protected neuronal function and improved learning and memory deficits caused by prolonged treatment (21 days) of 60 mg/kg dexamethasone using the Morris water maze task [58]. Thus, the present results are consistent with the earlier reports on the cognitive function and demonstrated the nootropic activity of ladies finger seeds.

Inhibition of acetylcholinesterase (AChE) is still considered as the main therapeutic strategy against Alzheimer's disease (AD) and cognitive deficits. Many plant derived phytochemicals claimed for nootropic activity have shown AChE inhibitory effect and/or antioxidant activity [59]. To our knowledge, based on thorough literature search, there was no report on AChE inhibitory effect of* Abelmoschus esculentus*. However, its major bioactive principles, quercetin, and rutin have been reported for AChE inhibitory effect [60]. Nasal administration of quercetin (in the form of liposomes, 0.5 mg of quercetin, once daily, 3 weeks) in a AF64A (ethylcholine mustard aziridinium) rat model of Alzheimer's disease improved memory deficits (spatial learning and memory) studied in Morris water maze test [61]. Further studies in this direction are currently underway in our laboratory to further explore the precise mechanism involved in the nootropic activity of* Abelmoschus esculentus* seed extracts.

In conclusion, the present study results provide evidence that the aqueous and methanolic seed extracts of* Abelmoschus esculentus* possess antioxidant, antistress (adaptogenic), and nootropic activities. This study also provides scientific evidence of the traditional claim of* Abelmoschus esculentus* fruits in stress-related disorders and dementia. Further investigations are required to characterize the active constituent(s) responsible for the observed activities and to elucidate the detailed mechanism of action at the cellular and molecular levels.

## Figures and Tables

**Table 1:** FTIR spectral peak values and functional groups obtained.

Extract	Peak values (cm^−1^)	Functional groups
Aqueous extract of *A. esculentus *	1375, 1454	C–H bending
	1638	C=C group
	1743	C=O carbonyl group
	2854, 2923	C–H stretching
	3285	–OH group

Methanolic extract of *A. esculentus *	1368, 1454	C–H bending
	1618	C=C group
	1742	C=O carbonyl group
	2360, 2691	C–H stretching
	2853, 2923	C–H stretching
	3284, 3379	–OH group

**Table 2:** Mean relative organ weights of mice ingested with 2000 mg/kg aqueous and methanolic seed extracts of *A. esculentus*. Each value is expressed as organ-to-body weight % ratio.

Organ	Control	AE	ME
Liver	6.93 ± 0.34	6.22 ± 0.29	6.34 ± 0.25
Heart	0.56 ± 0.05	0.46 ± 0.04	0.49 ± 0.05
Kidney	1.66 ± 0.18	1.49 ± 0.13	1.50 ± 0.14
Lungs	0.16 ± 0.09	0.15 ± 0.06	0.16 ± 0.03
Spleen	0.38 ± 0.04	0.35 ± 0.03	0.36 ± 0.04
Brain	1.98 ± 0.14	1.83 ± 0.11	1.89 ± 0.16

**Table 3:** Hematological parameters of mice ingested with 2000 mg/kg aqueous (AE) and methanolic (ME) seed extracts of *A. esculentus*.

Haematological parameter	Control	AE	ME
WBC (10^3^/mm^3^)	10.25 ± 0.72	18.1 ± 4.27	17.9 ± 2.28
Lymphocyte (%)	84 ± 0.52	78.6 ± 1.99	79.2 ± 0.86
Monocyte (%)	22.08 ± 0.27	16.9 ± 1.62	17.66 ± 1.23
Granulocytes (%)	13.28 ± 0.73	10.94 ± 1.77	10.04 ± 2.35
RBC (10^6^/mm^3^)	7.90 ± 0.77	7.45 ± 1.48	7.71 ± 0.98
Platelet (10^3^/mm^3^)	702.6 ± 2.12	684.5 ± 5.2	655 ± 13.84
MPV (*µ*m^3^)	8.8 ± 0.69	8.7 ± 1.93	9.1 ± 2.04
Hb (g/dL)	11.99 ± 0.47	12.3 ± 1.25	12.28 ± 1.54
Hematocrit (%)	31.9 ± 0.94	38.05 ± 4.47	42.5 ± 6.96
MCV (*µ*m^3^)	48 ± 1.61	52.5 ± 2.62	50.55 ± 6.62

Values are mean ± SD; *n* = 6, MCV: mean corpuscular volume; MPV: mean platelet volume, Hb: Hemoglobin.
